# Polygenic risk for obesity and its interaction with lifestyle and sociodemographic factors in European children and adolescents

**DOI:** 10.1038/s41366-021-00795-5

**Published:** 2021-03-22

**Authors:** Anke Hüls, Marvin N. Wright, Leonie H. Bogl, Jaakko Kaprio, Lauren Lissner, Dénes Molnár, Luis A. Moreno, Stefaan De Henauw, Alfonso Siani, Toomas Veidebaum, Wolfgang Ahrens, Iris Pigeot, Ronja Foraita

**Affiliations:** 1 Department of Epidemiology and Gangarosa Department of Environmental Health, Rollins School of Public Health, Emory University, Atlanta, GA USA; 2 Leibniz Institute for Prevention Research and Epidemiology—BIPS, Bremen, Germany; 3 Faculty of Mathematics and Computer Science, University of Bremen, Bremen, Germany; 4 Department of Epidemiology, Center for Public Health, Medical University of Vienna, Vienna, Austria; 5 Institute of Molecular Medicine FIMM, University of Helsinki, Helsinki, Finland; 6 Department of Public Health, University of Helsinki, Helsinki, Finland; 7 Department of Public Health and Community Medicine, Institute of Medicine, Sahlgrenska Academy, University of Gothenburg, Gothenburg, Sweden; 8 Department of Paediatrics, Medical School, University of Pécs, Pécs, Hungary; 9 GENUD (Growth, Exercise, Nutrition and Development) Research Group, University of Zaragoza, Instituto Agroalimentario de Aragón (IA2), Instituto de Investigación Sanitaria de Aragón (IIS Aragón), Zaragoza, Spain; 10 Centro de Investigación Biomédica en Red de Fisiopatología de la Obesidad y Nutrición (CIBEROBN), Instituto de Salud Carlos III, Madrid, Spain; 11 Faculty of Medicine and Health Sciences, Department of Public Health and Primary Care, Ghent University, Ghent, Belgium; 12 Institute of Food Sciences, CNR, Avellino, Italy; 13 Department of Chronic Diseases, National Institute for Health Development, Tallinn, Estonia

**Keywords:** Genetics, Risk factors

## Abstract

**Background:**

Childhood obesity is a complex multifaceted condition, which is influenced by genetics, environmental factors, and their interaction. However, these interactions have mainly been studied in twin studies and evidence from population-based cohorts is limited. Here, we analyze the interaction of an obesity-related genome-wide polygenic risk score (PRS) with sociodemographic and lifestyle factors for BMI and waist circumference (WC) in European children and adolescents.

**Methods:**

The analyses are based on 8609 repeated observations from 3098 participants aged 2–16 years from the IDEFICS/I.Family cohort. A genome-wide polygenic risk score (PRS) was calculated using summary statistics from independent genome-wide association studies of BMI. Associations were estimated using generalized linear mixed models adjusted for sex, age, region of residence, parental education, dietary intake, relatedness, and population stratification.

**Results:**

The PRS was associated with BMI (beta estimate [95% confidence interval (95%—CI)] = 0.33 [0.30, 0.37], *r*^2^ = 0.11, *p* value = 7.9 × 10^−81^) and WC (beta [95%—CI] = 0.36 [0.32, 0.40], *r*^2^ = 0.09, *p* value = 1.8 × 10^−71^). We observed significant interactions with demographic and lifestyle factors for BMI as well as WC. Children from Southern Europe showed increased genetic liability to obesity (BMI: beta [95%—CI] = 0.40 [0.34, 0.45]) in comparison to children from central Europe (beta [95%—CI] = 0.29 [0.23, 0.34]), *p-*interaction = 0.0066). Children of parents with a low level of education showed an increased genetic liability to obesity (BMI: beta [95%—CI] = 0.48 [0.38, 0.59]) in comparison to children of parents with a high level of education (beta [95%—CI] = 0.30 [0.26, 0.34]), *p*-interaction = 0.0012). Furthermore, the genetic liability to obesity was attenuated by a higher intake of fiber (BMI: beta [95%—CI] interaction = −0.02 [−0.04,−0.01]) and shorter screen times (beta [95%—CI] interaction = 0.02 [0.00, 0.03]).

**Conclusions:**

Our results highlight that a healthy childhood environment might partly offset a genetic predisposition to obesity during childhood and adolescence.

## Introduction

Obesity is a complex multifaceted condition and its prevalence has been increasing continuously over previous decades most likely due to adverse changes of environmental and demographic factors [1]. Studies in twins have suggested that genetic factors explain ~40–80% of the variation in obesity susceptibility [2]. Twin studies have further suggested that obesity-predisposing genes are not deterministic, but they rather interact with a variety of environmental and lifestyle factors. In particular, the heritability of BMI has been shown to be higher among children living in obesogenic home environments [3–6], children whose parents have lower education levels [7] and young adults with a sedentary lifestyle [8, 9]. An alternative to the traditional twin study design is genome-wide associations studies (GWAS), which have revolutionized the field of complex disease genetics over the past decade, providing numerous compelling associations for obesity [10, 11] and other human complex traits and diseases [12]. GWAS have identified 751 genetic variants (single-nucleotide polymorphisms (SNPs)) in association with BMI [10, 11] and a subset of them has been used in gene–environment (G×E) interaction analyses to show that the genetic predisposition to obesity is attenuated by a healthy lifestyle including physical activity [13, 14] and adherence to healthy dietary patterns [14–20]. However, these genome-wide significant variants only account for a small portion of BMI variation (up to 6%) [10, 11], while genome-wide estimates suggest that common variation accounts for >20% of BMI variation [10]. Therefore, the polygenic nature of BMI is not reflected in the current literature of BMI-related G×E interactions, which could have decreased the statistical power to detect interactions. Khera et al. suggest that the power to predict BMI can be improved by using polygenic risk scores (PRSs) that include SNPs that do not reach the threshold for genome-wide significance and by using genome-wide approaches [21]. We hypothesize that using a PRS that captures the polygenic nature of BMI will enable us to validate the interactions that were found in twin studies [3–9] and possibly detect new G×E interactions that have not been found by previous studies.

Another gap in knowledge is that most previous G×E interaction studies primarily involved adults [8, 9, 13–20, 22, 23], so that little is known whether the inherited susceptibility to obesity is modified by environmental factors already during childhood and adolescence. Given that the weight trajectories of individuals in different PRS deciles start to diverge in early childhood [21], the identification of robust G×E interactions in children is particularly important to facilitate targeted strategies for obesity prevention early in life.

In this study, we will calculate the most recent PRS for BMI [21] and (1) show the variance explained by the PRS for BMI as well as for waist circumference of European children and adolescents and (2) analyze its interaction with parental education, region of residence, selected dietary variables, and physical activity to investigate to which degree the inherited susceptibility to obesity in children is modified by these sociodemographic and lifestyle factors. The analyses are based on 8609 repeated observations from 3098 children and adolescents aged 2–16 years from the pan-European IDEFICS/I.Family cohort.

## Methods

### Study population

The pan-European IDEFICS/I.Family cohort [24, 25] is a multi-center, prospective study on the association of social, environmental, and behavioral factors with children’s health status. Children were recruited through kindergarten or school settings in Belgium, Cyprus, Estonia, Germany, Hungary, Italy, Spain, and Sweden. In 2007/2008, 16,229 children aged between 2 and 9.9 years participated in the baseline survey. Follow-up surveys were conducted after 2 (FU1, *N* = 11,043 plus 2543 newcomers) and 6 years (FU2, *N* = 7117 plus 2512 newly recruited siblings). Questionnaires were completed by parents. In the second follow-up (FU2), adolescents of 12 years of age or older reported for themselves. The study was conducted in agreement with the Declaration of Helsinki; all procedures were approved by the local ethics committees and written and oral informed consents were obtained. Children were selected for a whole-genome scan based on their participation in the individual study modules. Children from Cyprus were not included in this initial genotyping to minimize population stratification.

### Assessment of BMI and waist circumference

BMI was calculated as weight divided by height squared [kg/m²]. Height was measured to the nearest 0.1 cm by a SECA 225 Stadiometer (Seca GmbH & Co. KG., Hamburg, Germany) and body weight was measured in fasting state in light underwear on a calibrated scale accurate to 0.1 kg by a Tanita BC 420 SMA scale (TANITA, Tokyo, Japan). Waist circumference was measured in upright position with relaxed abdomen and feet together using an inelastic tape (Seca 200, Birmingham, UK), precision 0.1 cm, midway between the iliac crest and the lowest rib margin to the nearest 0.1 cm [26]. Age- and sex-specific BMI and waist circumference *z*-scores for children and adolescents were calculated using reference data from the International Obesity Task Force [27] and from British children [28], respectively. In addition, we proceeded as follows to dichotomize BMI and waist circumference (binary outcomes): As recommended by the International Obesity Task Force [27], we used age- and sex-specific cutoff values for obesity based on the raw BMI values, e.g., 6.0-year-old boys and girls with a BMI of at least 19.76 and 19.62 were considered as obese, respectively. The age- and sex-specific cutoff values for waist circumference were based on the top quartile of the reference data from the National Health and Nutrition Examination Survey [29], e.g., 6.0 year old boys and girls with a waist circumference of at least 58.3 and 57.2 cm were in the top quartile of waist circumference, respectively.

### Genotyping and quality control

DNA was extracted from saliva or blood samples using established procedures. Genotyping of 3515 children was performed on the UK Biobank Axiom array (Santa Clara, USA) in two batches (2015 and 2017). Following the recommendations of ref. [30], sample and genotype quality control measures were applied (see Supplementary materials for details), resulting in 3099 children and 3424,677 genotypes after imputation. A genetic relatedness matrix was calculated by using the program EMMAX (https://genome.sph.umich.edu/wiki/EMMAX) to account for the degree of relatedness within the study sample and to adjust for population stratification [31, 32] (see “Statistical analyses”).

### Polygenic risk score calculation

We calculated PRS based on genome-wide summary statistics for BMI from European ancestry populations. The PRS (called PRS-Khera) was proposed and validated in Khera et al. [21]. It consists of 2,100,302 SNPs and is based on summary statistics from the first large-scale GWAS of BMI (~300,000 samples) [10]. PRS-Khera was calculated in Khera et al. [21] using a computational algorithm called LDPred, which is a Bayesian approach to calculate a posterior mean effect for all variants using external weights with subsequent shrinkage based on linkage disequilibrium [33]. Using LDPred, each variant was reweighted according to the prior GWAS [10], the degree of correlation between a variant and others nearby, and a tuning parameter that denotes the proportion of variants with non-zero effect.

In sensitivity analyses, the performance of PRS-Khera was compared to PRS calculated with PRSice [34] and PRS based on only genome-wide significant SNPs from two discovery samples (same discovery sample as for PRS-Khera (~300,000 samples) [10] and the largest published GWAS study of BMI to date (~700,000 samples) [11]). More details on the different PRS are given in the Supplementary methods and Figs. S1–S3.

### Assessment of dietary intake

We used long-term and short-term dietary measurements assessed by food frequency questionnaires (FFQs) and repeated 24-h dietary recalls, respectively [35]. A fruit and vegetable score was calculated from FFQs (for more details on the FFQs and calculation of the fruit and vegetable score, see Supplementary material). We expressed the fruit and vegetable consumption as the relative frequency in relation to all foods reported in the FFQs [36]. Energy and dietary fiber intake was assessed by repeated 24-h dietary recalls in a subset of the IDEFICS/I.Family cohort (see Table 1 for the actual numbers) [37, 38]. Fiber intake was expressed in relation to total energy intake in mg/kcal. See Supplementary material for more details.

**Table 1:** Study characteristics of the 8609 repeated observations from 3098 children.

	Baseline	First follow-up (FU1)	Second follow-up (FU2)
*n*	3016	2937	2656
Age (years)			
Mean (SD)	6.19 (1.77)	8.12 (1.80)	11.75 (1.83)
Median (IQR)	6.60 (3.10)	8.50 (3.20)	11.90 (3.20)
Range	2.0–9.7	3.4–11.9	6.6–16.2
Sex			
Female (%)	1510 (50.07)	1472 (50.12)	1331 (50.11)
Male (%)	1506 (49.93)	1465 (49.88)	1325 (49.89)
Parental education			
Low (%)	180 (5.97)	166 (5.65)	156 (5.87)
Medium (%)	1337 (44.33)	1204 (40.99)	1172 (44.13)
High (%)	1463 (48.51)	1476 (50.26)	1310 (49.32)
Missing	36	91	18
European region of residence			
Central (%)	1250 (41.45)	1218 (41.47)	1114 (41.94)
North (%)	743 (24.64)	721 (24.55)	682 (25.68)
South (%)	1023 (33.92)	998 (33.98)	860 (32.38)
Fruit and vegetable score (%)			
Mean (SD)	14.66 (7.49)	15.39 (7.99)	14.68 (7.83)
Median (IQR)	13.80 (9.58)	14.68 (10.24)	13.68 (9.66)
Range	0.00–57.14	0.00–58.33	0.00–60.71
Missing	58	154	106
Fiber intake (mg/kcal)			
Mean (SD)	8.17 (1.31)	8.23 (0.90)	8.22 (1.27)
Median (IQR)	8.13 (1.79)	8.13 (1.48)	8.07 (1.61)
Range	3.87–15.76	5.76–11.56	4.74–13.89
Missing	826	1100	660
MVPA (h/day)			
Mean (SD)	0.67 (0.36)	0.67 (0.36)	0.64 (0.37)
Median (IQR)	0.61 (0.46)	0.62 (0.47)	0.57 (0.47)
Range	0.02–2.29	0.03–2.74	0.00–2.42
Missing	1240	1297	871
Screen time (h/day)			
Mean (SD)	1.60 (1.00)	1.89 (1.08)	2.34 (1.50)
Median (IQR)	1.50 (1.07)	1.75 (1.43)	2.02 (1.79)
Range	0.00–8.00	0.00–8.00	0.00–8.00
Missing	93	132	150
BMI *z*-scores			
Mean (SD)	0.34 (1.16)	0.41 (1.18)	0.51 (1.12)
Median (IQR)	0.23 (1.48)	0.32 (1.67)	0.45 (1.62)
Range	−5.42–5.80	−5.76–4.65	−2.96–3.83
Obese (%)	204 (6.76)	214 (7.29)	179 (6.74)
Waist circumference *z*-scores			
Mean (SD)	0.24 (1.45)	0.59 (1.29)	0.78 (1.25)
Median (IQR)	0.16 (1.61)	0.46 (1.72)	0.71 (1.77)
Range	−27.98–5.65	−6.79–5.33	−7.75–4.38
Top quartile (%)	461 (15.29)	443 (15.08)	316 (11.90)
Missing	76	22	55

*Z*-scores for BMI and waist circumference were calculated according to refs. [27, 28]. Obesity was defined according to the extended IOTF criteria [27].

### Assessment of physical activity

Physical activity was objectively measured by using Actigraph’s uniaxial or three-axial accelerometers [39, 40]. At baseline and FU1, children were asked to wear the accelerometer for 3 days (including 1 weekend day) and at FU2 for a full week during waking hours (except when swimming or showering). The daily average cumulative duration of time spent performing moderate-to-vigorous physical activity (MVPA) was expressed as hours per day according to the cutoff value by Evenson et al. [41]. Time spent in MVPA is based on cleaned accelerometer data that only contain measurements that have passed the minimum wear time criteria of at least 3 measurement days and at least 360 min of valid time per day. The accelerometers were attached to the right hip with an elastic belt. See Supplementary material for more details.

### Assessment of screen time

Screen time was assessed by asking how many hours per day the child/adolescent usually spends watching television (including videos or DVDs) and by another question on the time sitting in front of a computer and game console [42, 43]. Responses were weighted and summed across weekdays and weekend days and the quantified frequencies from both questions were added to create a continuous variable of total screen time in hours per day. See Supplementary material for more details.

### Assessment of sociodemographic variables

Parental education was retrieved from questionnaires and coded according to the International Standard Classification of Education (ISCED) [44]. For the analyses, the highest parental education of both parents was coded as low (ISCED levels 1 and 2; ≤9 years of education), medium (ISCED levels 3 and 4), and high (ISCED levels 5 and 6; ≥2 years of education after high school). The region of residence was coded as Northern Europe (Estonia, Sweden), Central Europe (Belgium, Germany, and Hungary), and Southern Europe (Italy, Spain).

### Statistical analyses

Our data consist of up to three repeated measurements of individuals, some of whom were siblings. We estimated associations between the PRS and obesity outcomes (BMI and waist circumference) as well as interactions between the PRS and demographic and lifestyle factors using generalized linear mixed models where the covariance matrix of the random intercept is proportional to a genetic relatedness matrix. We applied the generalized linear mixed model approach of Chen et al. [31] that jointly controls for relatedness and population stratification. Such a model can be formulated in slightly simplified notation as:

$$g\left( {E(y)} \right)=X\beta + \gamma$$

$$\gamma \sim N\left( {0,V} \right),$$

where *g*() is the link function, *E*() the expectation, *y* is the dependent variable, *X* the covariate matrix, *β* a vector of the fixed effects, and *γ* the intercept-only random effect, which is assumed to be normally distributed with expectation 0 and covariance according to the genetic relatedness matrix *V*.

In addition, we conducted the following analyses for the main effects of the PRS for easier interpretation and comparison with the results from Khera et al. [21]. (1) We used logistic mixed models (logit link) to estimate associations between the PRS and obesity and the top quartile of waist circumference (binary outcomes) and (2) we estimated associations between being in the top decile of the PRS (binary variable) and the obesity outcomes.

All models were adjusted for confounding factors that are assumed to be associated with lifestyle and obesity (sex, age, region of residence, parental education, and dietary intake (fruit and vegetable score as proxy for healthy dietary intake)). Models that investigated the interaction between PRS and fiber intake were not additionally adjusted for the fruit and vegetable score because both variables are used as proxy variables for healthy dietary intake. The response and confounding variables showed only small percentage of missing values while we had more missing values of some exposure variables such as fiber intake and MVPA (Table 1). We compared BMI and waist circumference of children with and without missing values in exposure variables (fiber, fruit and vegetable score, MVPA, screen time) to evaluate if they were missing at random. As we conducted a repeated measurement analysis, we retained all children in the analysis that had at least one observed measurement of each variable and performed listwise deletion of incomplete cases. When testing associations with categorical variables (sex, region of residence, and parental education), we used the category with the largest sample size as reference category.

All *p* values from the G×E interaction analyses were adjusted according to the number of tested environmental factors using the false-discovery rate (FDR, FDR-adjusted *p* values are called *q* values). We reported 95% CI and two-sided *p* values, and considered *p* values <0.05 statistically significant. We used R 3.5.1 [45] for all statistical analyses.

## Results

### Study description

The study sample included 8609 repeated BMI measurements from at maximum three time points (baseline, FU1, FU2) of 3098 children aged 2–16 years (Table 1). The number of participants decreased between the follow-up investigations from *n* = 3016 at baseline (mean age 6 years) to *n* = 2656 at FU2 (mean age 12 years). Half of the children were girls, most children came from families with a medium or high level of parental education and the majority lived in Central European countries. The distributions of the dietary variables (fruit and vegetable score and fiber intake) and time spent in MVPA were similar between baseline and the two follow-up samples, whereas children and adolescents spent more time in front of screens at FU1 and FU2 as compared to baseline. For the variables with the most missing values (MVPA, fiber intake, the fruit and vegetable score, and screen time), we observed at least one of three repeated measurements for 90%, 95%, >99%, and >99% of the children, respectively. We found no substantial differences between children with no measurements at any visit and children with at least one observed measurement with BMI, waist circumference, and the PRS score (see Fig. S4).

### Variance explained by PRS

We found that the PRS-Khera provided the best prediction of BMI (*r*^2^ = 0.11) and the second-best prediction of obesity (AUC = 0.74, see Table S1 for details on the characteristics of the other PRS). PRS-Khera was associated with BMI (*r*^2^ = 0.11, *p* value = 7.9 × 10^−81^) and waist circumference (*r*^2^ = 0.09, 1.8 × 10^−71^) in our study population (Table 2) and these correlations increased with age (see Tables S2, S3 and Fig. S5). Being in the top decile of the distribution of PRS-Khera was associated with 3.63 times higher odds for obesity (95% CI: [2.57, 5.14]) and with 3.09 (95% CI: [2.37, 4.03]) higher odds for being in the top quartile of waist circumference.

**Table 2:** Associations of PRS-Khera with BMI, obesity, and waist circumference in IDEFICS/I.Family.

(A) BMI						
	BMI			Obesity		
Scale of PRS	Est., 95% CI	*p* value	*R*²	OR, 95% CI	*p* value	AUC
Continuous	0.33 [0.30, 0.37]	7.9e − 81	0.108	2.33 [2.01, 2.70]	2.0e − 29	0.736
Top decile	0.61 [0.49, 0.73]	5.4e − 24	0.036	3.63 [2.57, 5.14]	2.7e − 13	0.598

(B) Waist circumference						
	Waist circumference			Waist top quartile		
Scale of PRS	Est., 95% CI	*p* value	*R*²	OR, 95% CI	*p* value	AUC
Continuous	0.36 [0.32, 0.40]	1.8e − 71	0.088	1.97 [1.78, 2.17]	1.5e − 40	0.683
Top decile	0.69 [0.55, 0.82]	8.8e − 24	0.032	3.09 [2.37, 4.03]	6.1e − 17	0.569

Associations adjusted for region of residence, sex, age, parental education, fruit and vegetable score. *Z* scores for BMI and waist circumference were calculated according to refs. [27, 28]. Obesity was defined according to the extended IOTF criteria [27].

### G×E interactions

**Fig. 1:**
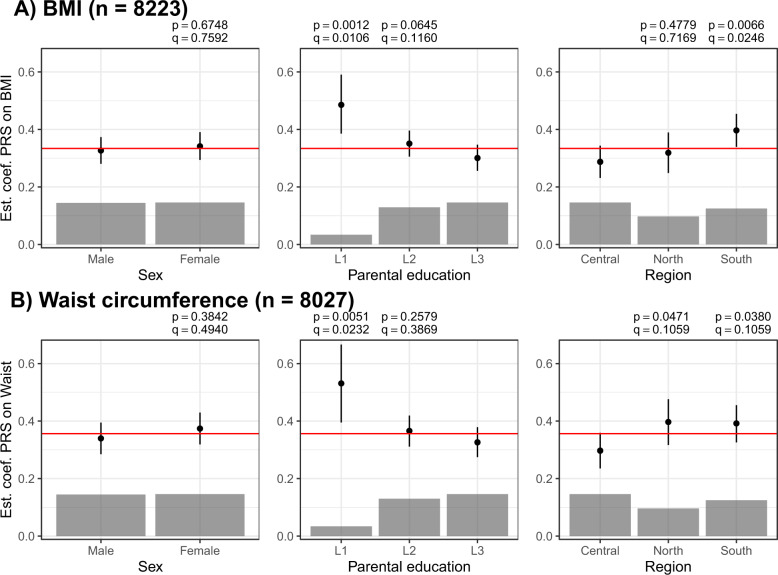
Interactions between PRS-Khera and sociodemographic factors on BMI and waist circumference. Associations between PRS and BMI/waist circumference are shown in different strata (beta estimates and 95% CIs) as well as in the whole study population (red line). Raw *p* values (*p*) and FDR-adjusted *p* values (*q* values, *q*) are given for the test of deviations of the association between PRS and obesity in one subgroup in comparison to the reference category (interaction). The category without *p* values is the reference category. The gray boxes show the distribution of the sociodemographic factors.

We found a significant G×E interaction of PRS-Khera with parental education (low vs. high) as well as with the European region of residence (Central vs. Southern) for BMI as well as for waist circumference (Fig. 1 and Table S4). Children and adolescents from families with a low level of parental education were at a higher risk of having obesity among those with higher genetic susceptibility than children from families with a high level of parental education (low: beta estimate from education-stratified analysis for association between PRS-Khera and BMI = 0.48; 95% CI: [0.38, 0.59], high: beta estimate = 0.30; 95% CI: [0.26, 0.34], *q* value interaction = 0.0106, Fig. 1 and Table S4). Furthermore, children and adolescents from Southern European countries showed an increased genetic susceptibility to a high BMI in comparison to children and adolescents from Central Europe (Central Europeans: beta estimate from region-stratified analysis for association between PRS-Khera and BMI = 0.29; 95% CI: [0.23, 0.34], Southern Europeans: beta estimate = 0.40; 95% CI: [0.34, 0.45], *q* value interaction = 0.0246, Fig. 1 and Table S4). Interactions were confirmed in our sensitivity analyses using other genome-wide PRS (Fig. S6 and Table S6). We did not find significant interactions between PRS-Khera and sex, the comparison of low vs. medium parental education, nor the comparison of Central vs. Northern European region of residence (Fig. 1 and Table S4).

**Fig. 2:**
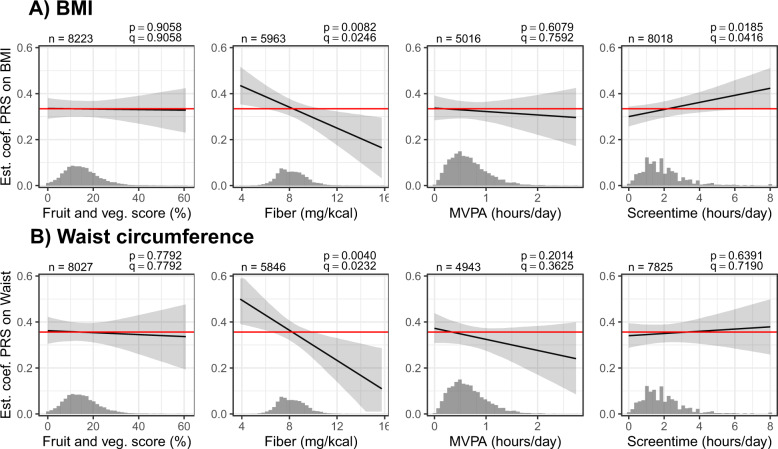
Interactions between PRS-Khera and lifestyle factors on BMI and waist circumference. Associations between PRS and obesity are shown in dependence of the PRS (beta estimates and 95% CIs) as well as in the whole study population (red line). The distributions of the lifestyle factors are shown in histograms. Raw *p* values (*p*) and FDR-adjusted *p* values (*q* values, *q*) are given for the interaction terms. The distributions of the lifestyle factors are shown in histograms.

The genetic susceptibility to a high BMI was further modified by intake of dietary fiber and screen time (Fig. 2 and Table S5). Children and adolescents with a higher fiber intake showed an attenuated risk of having obesity despite their genetic susceptibility (BMI: beta estimates and 95% CI for interaction terms: −0.02 [−0.04, −0.01], *q* values interaction = 0.025; waist circumference: −0.03 [−0.06, −0.01], *q* values interaction = 0.023). Furthermore, the more time the children and adolescents spent in front of screens, the higher was their risk of having obesity among those with higher genetic susceptibility (significant for BMI: beta estimates and 95% CI for interaction terms: 0.02 [0.00, 0.03], *q* value interaction = 0.042). Interactions between PRS-Khera and the fruit and vegetable score or MVPA were not significant (beta estimates and 95% CI for interaction terms: −0.01 [−0.21, 0.19] for fruit and vegetable score and −0.01 [−0.07, 0.04] for MVPA). Interaction results with other PRS for obesity were similar, but not significant (Fig. S7 and Table S7).

## Discussion

In our pan-European cohort of children aged 2–16 years, we found significant interactions between PRS-Khera and sociodemographic as well as lifestyle factors for BMI and waist circumference: we observed G×E interactions with (1) the European region of residence, which most likely reflect cultural lifestyle differences, (2) parental education, (3) dietary fiber intake, and (4) the time children spent in front of screens. Of note, all of these interactions would have remained undetected in this sample of children when only focusing on genome-wide significant variants as was done in previous studies (compare Figs. S6 and S7) [13–20].

### Comparison with previous studies

Interactions with socioeconomic status [7, 14], physical activity [8, 9, 13, 14], and dietary factors [14–16] have been reported previously. However, previous interaction results were mainly estimated in twin studies, which might not be representative of the general population [46], and cohort studies including only <100 genome-wide significant SNPs, which do not account for the polygenic nature of BMI [21]. Thus, our study confirms previous interaction findings and demonstrates that genome-wide PRSs are a powerful approach to detect interactions and a good alternative to the traditional twin study design. Genome-wide PRSs have the advantage that they can be applied to cohort studies, while explaining a much larger part of the genetic variance of BMI than studies restricted to genome-wide significant variants. In addition, previous G×E interaction studies were mainly based on adult populations whereas in our study we analyzed data from children and adolescents aged 2–16 years, i.e., in the key developmental transition phases of human life.

We identified children from families with low level of parental education as being about 61% more susceptible to the polygenic burden of obesity than children from families with a high level of parental education. In addition, we found that children from Southern Europe had a higher genetic susceptibility to obesity in comparison to children from Central Europe. Parental education and region of residence reflect a variety of social and cultural differences and many of them are difficult to be captured by questionnaires. Since a previous analysis of the same cohort showed that low parental education was associated with higher intakes of unhealthy food among children, e.g., sugar-rich and fatty foods [47, 48], part of the effect modification might be due to dietary habits. The differences in the risk of having obesity among children with a higher genetic susceptibility across different European regions might be explained by differences in dietary or cultural habits [49, 50].

Furthermore, we found an interaction between PRS-Khera and dietary fiber intake, where children with a higher intake of fiber have a reduced risk for obesity despite their genetic susceptibility. This finding is in line with many other studies that have shown that a healthy diet can attenuate the genetic burden of obesity [14–20].

Interactions between PRS-Khera and physical activity (MVPA) were not significant, but the direction of interaction effect was in line with previous studies [13, 14]. An explanation for this might be that MVPA was only assessed in ~40% of our analysis group (Table 1), which reduced the statistical power to detect interactions between MVPA and PRS.

### Strengths and limitations of this study

Important strengths of this study include: detailed and repeated phenotyping of participants in this cohort with partly objective measures (MVPA), inclusion of thousands of children from diverse regions in Europe and the longitudinal approach across key developmental periods [25]. Dietary assessment in children is a challenging task, and different dietary assessment methods have different strengths and limitations. We used two different methods—a fruit and vegetable score derived from FFQs and fiber intake calculated from the more detailed 24-h dietary recalls. The harmonized protocol in all countries that was enforced by a central quality control and a central data management ensures comparability of measurements across study centers. Another major strength of our study is the application of genome-wide PRS for obesity, which has an almost five times higher prediction accuracy than previously used PRS [14–20] and with which we identified interactions that would have remained undetected when restricting to only genome-wide significant variants (compare Figs. S5 and S6). In addition, although the PRS-Khera was derived for BMI we also assessed its association with waist circumference. The strength of this association was only slightly smaller than the association with BMI. This is plausible, because PRS-Khera is known to be a strong risk factor for severe obesity and associated health outcomes [21].

A limitation of our study is that measurement errors of self-reported lifestyle behaviors are inevitable. However, measurement error in environmental exposure typically biases the interaction effect toward the null [51], which does not increase the risk for false-positive findings but reduces the statistical power to detect modest interactions. In addition, we used a complete-case analysis strategy, which might bias the estimates toward null [52].

## Conclusions

Our study showed significant interactions between the polygenic risk for an increased BMI and sociodemographic and lifestyle factors that affect BMI as well as waist circumference. Among children with a high genetic risk, we identified children from Southern Europe, children from families with a low level of parental education, children with a low dietary fiber intake and children who spend more time in front of screens as being particularly susceptible to obesity. These results suggest that the risk for obesity among children with a high genetic susceptibility varies by environmental and sociodemographic factors during childhood. While all children benefit from an environment that supports a healthy lifestyle, our findings suggest that this is particularly important for children with a high genetic risk for obesity. Although it is unlikely that genetic screening for obesity will be implemented in clinical practice anytime soon, our findings emphasize the importance of obesity prevention in early childhood by showing that there are synergistic effects of genetics and sociodemographic and lifestyle factors that could affect a substantial part of the general population. The interactions between parental education, region, and genetic heritability indicate that system-level interventions might be better suited than individual intervention strategies.

## Supplementary information

Supplemental Material
