# Supplementary material for: Polygenic risk for obesity and its interaction with lifestyle and sociodemographic factors in European children and adolescents
Source: Int J Obes (Lond). 2021 Mar 22;45(6):1321–30. doi: 10.1038/s41366-021-00795-5 (PMC8159747; doi:10.1038/s41366-021-00795-5)
Supplement: Supplementary file 1 — Supplemental Material [file 41366_2021_795_MOESM1_ESM.pdf]

Supplementary Material

Polygenic risk for obesity and its interaction with lifestyle and socio-demographic factors in European children and adolescents

Anke Hüls*[1], Marvin N Wright*[2,13], Leonie H Bogl[3,4], Jaakko Kaprio[4,5], Lauren Lissner[6], Dénes Molnár[7], Luis A Moreno[8,9], Stefaan De Henauw[10], Alfonso Siani[11], Toomas Veidebaum[12], Wolfgang Ahrens[2,13], Iris Pigeot[#2,13], Ronja Foraita[#2]

[1] Department of Epidemiology and Gangarosa Department of Environmental Health, Rollins School of Public Health, Emory University, Atlanta, Georgia, USA

[2] Leibniz Institute for Prevention Research and Epidemiology – BIPS, Bremen, Germany

[3] Department of Epidemiology, Center for Public Health, Medical University of Vienna, Austria

[4] Institute of Molecular Medicine FIMM, University of Helsinki, Helsinki, Finland

[5] Department of Public Health, University of Helsinki, Helsinki, Finland

[6] Department of Public Health and Community Medicine, Institute of Medicine, Sahlgrenska Academy, University of Gothenburg, Gothenburg, Sweden

[7] Department of Paediatrics, Medical School, University of Pécs, Pécs, Hungary

[8] GENUD (Growth, Exercise, Nutrition and Development) Research Group, University of Zaragoza, Instituto Agroalimentario de Aragón (IA2), Instituto de Investigación Sanitaria de Aragón (IIS Aragón), Zaragoza, Spain

[9] Centro de Investigación Biomédica en Red de Fisiopatología de la Obesidad y Nutrición (CIBEROBN), Instituto de Salud Carlos III, Madrid, Spain

[10] Department of Public Health and Primary Care, Faculty of Medicine and Health Sciences, Ghent University, Ghent, Belgium

[11] Institute of Food Sciences, CNR, Avellino, Italy

[12] Department of Chronic Diseases, National Institute for Health Development, Tallinn, Estonia

[13] University of Bremen, Faculty of Mathematics and Computer Science, Bremen, Germany

[*,#] equal contribution

Methods

Genotyping and Quality Control

DNA was extracted from saliva or blood samples using established procedures. Genotyping of 3,515 children was performed on the UK Biobank Axiom array (Santa Clara, USA) in two batches (2015 and 2017). Following the recommendations of [1], sample and genotype quality control measures were applied. After visual inspection of the cumulative SNP coverage distribution, SNPs with call rates less than 97.5%, relative minor allele frequency (MAF) < 0.05 (batch1) and 0.08 (batch2), not being in Hardy-Weinberg equilibrium (p-value < 10^{-4}.) were excluded. Children with call rates less than 98% (batch1) and 96% (batch2), sex mismatch, anomalously high heterozygosity [1], duplicates, or population outliers were also excluded from the sample. We performed principal components analysis (PCA) to identify population outliers using the R package SNPRelate [2]. A sample of 3,099 children and 570,587 (batch1) / 551,909 (batch2) SNPs, respectively, remained for further analyses. Genome-wide imputation was conducted using Minimac3 (https://genome.sph.umich.edu/wiki/Minimac3) and reference haplotypes from unrelated individuals from the 1000 Genomes Project phase III v5. A total of 3,424,677 imputed genotypes with an estimated posterior genotype probability > 0.8 and MAF ≥ 0.05 from 3,099 children and adolescents were used in the analyses.

Polygenic Risk Score Calculation

In our sensitivity analyses, we calculated four PRS for BMI - two PRS based on genome-wide summary statistics of European ancestry populations and two based on only genome-wide significant SNPs from the discovery samples.

Two PRS were calculated using PRSice [3], one using the same discovery sample as for PRS-Khera (Locke^{BT}; BT: best threshold) and a second one based on summary statistics from the

largest published GWAS study of BMI to date (~700,000 samples) [4] (Yengo^{BT}). PRSice calculates the sum of alleles weighted by their effect sizes estimated from a GWAS of that phenotype in an independent sample (here from [4]) using an approach called clumping and thresholding. Clumping was used to obtain SNPs in linkage equilibrium with an $r^2 < 0.1$ within a 500 kbp window, keeping the SNP with the lower p-value observed in the external dataset for the analysis. Multiple scores were then created according to the significance of their association with the phenotype. As proposed in [3], the PRS that predicted the phenotype (BMI) best (highest R^2) was used for analysis, resulting in a p-value threshold of 1 for Locke^{BT} (Figure S1) and 0.5 for Yengo^{BT} (Figure S2).

The two PRS that were based on only genome-wide significant (GS) SNPs from the discovery samples (Locke^{GS} with significant SNPs from [5] and Yengo^{GS} with significant SNPs from [4]) were also calculated using PRSice by setting a fixed p-value threshold of 5×10^{-8}.

Assessment of Dietary Intake

Information on dietary intake was collected by two retrospective methods, repeated 24-hour dietary recalls (24HDRs) and food frequency questionnaires (FFQs). For the present analysis, fruit and vegetable intake was obtained from the FFQs, which were self-reported for adolescents 12 years and older and proxy-reported by a parent or other caregiver for children below the age of 12 years. In the IDEFICS study, the FFQ asked for the usual consumption frequency of 43 food and beverage items, out of which 4 were fruit and vegetable items including cooked vegetables, potatoes and beans; raw vegetables (mixed salad, carrot, fennel, cucumber, lettuce, tomato, and local examples); fresh fruits (also freshly squeezed, fruit smoothie) without added sugar; and fresh fruits (also freshly squeezed, fruit smoothie) with added sugar. In the I.Family study, the FFQ was extended to include 59 items for children and 60 item for adolescents, out of which 5 were fruit and vegetables items including potatoes

(cooked, not fried); other cooked vegetables (with local examples); raw vegetables (mixed salad, carrot, fennel, cucumber, lettuce, tomato, and local examples); fresh fruits (also as freshly squeezed juice) without added sugar; and fresh fruits (also as freshly squeezed juice) with added sugar. The FFQs in the IDEFICS/I.Family studies asked for the usual consumption frequency by the following question: “In the last month, how many times did you eat or drink the following food items?” Possible response categories were recoded to weekly consumption frequencies as follows: never/less than once a week (recoded to 0), 1–3 times a week (recoded to 2), 4–6 times a week (recoded to 5), 1 time per day (recoded to 7), 2 times per day (recoded to 14), 3 times per day (recoded to 21) or 4 or more times per day (recoded to 30). To maintain comparability across countries, the same foods and beverages were translated into eight languages, but for some food items, country-specific foods were also noted as examples. The items from the FFQ have been found to be reproducible with a weighted kappa coefficients of 0.49 and a Spearman's correlation coefficient of 0.59 for vegetables [6], and a validation study of the FFQ against repeated 24HDRs showed that the gross misclassification for fruit and vegetables was below 5% [7]. We expressed fruit and vegetable consumption as relative frequency of all consumed foods in the FFQ [8]. Items of the FFQ that were omitted were assumed as not consumed if less than half of the items on the FFQ were missing; otherwise, the consumption frequency was set to missing [8,9].

Energy and dietary fiber intake were assessed by using repeated 24HDRs [10,11]. The offline software called SACINA (Self-Administered Children and Infant Nutrition Assessment) used in the IDEFICS study was amended and delivered as a web-based version, called SACANA (Self-Administered Child, Adolescent and Adult Nutrition Assessment) in the I.Family study. In the IDEFICS study, parents or other caregivers proxy-reported 24HDRs for their children. In I.Family, children from 8 years of age onwards self-completed their 24HDRs, while parental assistance was given at younger ages. Participants were assisted by trained survey personnel

or dieticians when completing the first 24HDR and asked to recall and enter all foods and beverages (types and amounts) consumed during the previous day, starting with the first meal in the morning after waking up. Standardized photographs were displayed on the screen to assist portion size estimation. Food items from SACINA and SACANA were linked with country-specific food composition tables to calculate energy and nutrient intakes. Usual intakes for fiber were estimated based on the validated National Cancer Institute (NCI) method, which is one of the most widely accepted methods for this purpose [12,13]. This method allows for the inclusion of covariates such as age and accounts for different intakes on weekend days vs. weekdays, and further corrects for the day-to-day variation in energy and fiber intakes. Usual intakes were estimated for each child stratified by sex and considering age as a covariate. Fiber intake was here expressed in relation to total energy intake in mg/kcal.

Assessment of Physical Activity

Physical activity was objectively measured by using Actigraph's uniaxial or three-axial accelerometers [14]. At baseline and FU1, children were asked to wear the accelerometer for 3 days (including one weekend day) and at FU2 for a full week during waking hours (except when swimming or showering), so that a full day of physical activity could be assessed. The accelerometers were attached to the right hip with an elastic belt. Participants (either the parents or the adolescents themselves) were given written instructions on how to use the accelerometer and were asked to complete diaries to record non-wear times of the device. The daily average cumulative duration of time spent performing moderate-to-vigorous physical activity (MVPA) was expressed as hours per day according to the cut-off value by Evenson et al. [15]. Time spent in MVPA is based on cleaned accelerometer data that only contain measurements that have passed the minimum wear time criteria of at least 3 measurement days and at least 360 minutes of valid time per day. Although it does not cover all waking hours, a monitoring period of 360 minutes or more has been shown to provide an acceptable reliability

in young children [16,17]. The valid wearing time of the included sample was longer in practice (mean 12.0 (standard deviation (SD) 1.6) h/d). All accelerometer recordings were integrated over 60 s epochs. Non-wear time was defined as 20 min or more of consecutive 0 counts. The average activity level of the children was defined by counts per minute (cpm). The average activity level of the children was defined by counts per minute per day (cpm/d, average over all valid days).

Assessment of Screen Time

As a proxy indicator for sedentary time, screen time duration was assessed by asking how many hours per day the child/adolescent usually spends watching television (including videos or DVDs) and by another question on the time sitting in front of a computer and game console [18,19]. The first question was included in a pilot study testing the reproducibility of the questionnaire, and the results suggested agreement between repeat measures (Cohen's kappa: k = 0.85 (95% CI: [0.78,0.91]) [20]. For both questions, separate answers were given for weekdays and weekend days. The answer categories 'Not at all', 'less than 30 min. per day', '30 min. to 1 hour per day', 'about 1-2 hours per day', 'about 2-3 hours per day' and 'more than 3 hours per day' were recoded to daily frequencies with the conversion factors 0, 0.25, 0.75, 1.5, 2.5 and 4, respectively. Responses were weighted and summed over weekdays and weekend days and the quantified frequencies from both questions were added to create a continuous variable of total screen time in hours per day. Parents proxy-reported for their children at baseline and FU1, and for children <12 at FU2, while children ≥ 12 at FU2 self-reported their screen time.

Comparison of PRS-Khera with other PRS for BMI

The three PRS that were based on genome-wide summary statistics (Khera, Locke^{BT} and Yengo^{BT}) were moderately correlated (Spearman correlation between 0.61 and 0.66), whereas correlations with the PRS that were based on only genome-wide significant SNPs (Locke^{GS} and Yengo^{GS}) were much lower (0.16 to 0.56) (Figure S3).

We found that the PRS-Khera provided the best prediction of BMI (r^2=0.11, p-value = 7.9 x 10^{-81}) (Table 2). These associations were very robust across different discovery samples (300,000 samples from [5] versus 700,000 samples from [4]) and across different genome-wide PRS approaches (PRSice vs. LDPred) (Table S1). In contrast, previous PRS for BMI, that were only based on the 77 genome-wide significant SNPs from [5], explained a much smaller part of the variance of BMI (Locke^{GS}: r^2=0.02, p-value = 4.2 x 10^{-29}). Furthermore, the association strongly depended on the sample size of the discovery sample. Using the genome-wide significant SNPs from the 700,000 samples from [4] instead of the 300,000 samples from [5], increased the prediction accuracy of both BMI and waist circumference from r^2 = 0.02 to 0.06 for BMI and from r^2 = 0.02 to 0.05 for waist circumference.

The increase in correlations between PRS and BMI by age was very robust across different genome-wide PRS approaches (Khera, Locke^{BT}, Yengo^{BT}), but less pronounced for the PRS that were only based on genome-wide significant SNPs (Locke^{GS} and Yengo^{GS}) (Figure S4 and Table S3). The squared correlation of Locke^{GS} with BMI was about the same for every age group from r^2 = 0.01 [0.00, 0.03] to r^2 = 0.03 [0.00, 0.07] and the squared correlation of Yengo^{GS} with BMI increased from r^2 = 0.05 [0.02, 0.11] to r^2 = 0.11 [0.04, 0.17].

References

1 Weale ME. Quality control for genome-wide association studies. *Methods Mol Biol* 2010; **628**: 341–72.

2 Zheng X, Levine D, Shen J, Gogarten SM, Laurie C, Weir BS. A high-performance computing toolset for relatedness and principal component analysis of SNP data. *Bioinformatics* 2012; **28**: 3326–3328.

3 Euesden J, Lewis CM, O'Reilly PF. PRSice: Polygenic Risk Score software. *Bioinformatics* 2015; **31**: 1466–1468.

4 Yengo L, Sidorenko J, Kemper KE, Zheng Z, Wood AR, Weedon MN *et al.* Meta-analysis of genome-wide association studies for height and body mass index in ~700 000 individuals of European ancestry. *Hum Mol Genet* 2018; **27**: 3641–3649.

5 Locke AE, Kahali B, Berndt SI, Justice AE, Pers TH, Day FR *et al.* Genetic studies of body mass index yield new insights for obesity biology. *Nature* 2015; **518**: 197–206.

6 Lanfer A, Hebestreit A, Ahrens W, Krogh V, Sieri S, Lissner L *et al.* Reproducibility of food consumption frequencies derived from the children's eating habits questionnaire used in the IDEFICS study. *Int J Obes* 2011; **35**: S61–S68.

7 Bel-Serrat S, Mouratidou T, Pala V, Huybrechts I, Börnhorst C, Fernández-Alvira JM *et al.* Relative validity of the Children's Eating Habits Questionnaire-food frequency section among young European children: The IDEFICS Study. *Public Health Nutr* 2014; **17**: 266–276.

8 Arvidsson L, Bogl LH, Eiben G, Hebestreit A, Nagy P, Tornaritis M *et al.* Fat, sugar and water intakes among families from the IDEFICS intervention and control groups: First observations from I.Family. *Obes Rev* 2015; **16**: 127–137.

9 Hansson LM, Galanti MR. Diet-associated risks of disease and self-reported food consumption: how shall we treat partial nonresponse in a food frequency questionnaire? *Nutr Cancer* 2000; **36**: 1–6.

10 Intemann T, Pigeot I, De Henauw S, Eiben G, Lissner L, Krogh V *et al.* Urinary sucrose and fructose to validate self-reported sugar intake in children and adolescents: results from the I.Family study. *Eur J Nutr* 2019; **58**: 1247–1258.

11 Bogl LH, Silventoinen K, Hebestreit A, Intemann T, Williams G, Michels N *et al.* Familial resemblance in dietary intakes of children, adolescents, and parents: Does dietary quality play a role? *Nutrients* 2017; **9**. doi:10.3390/nu9080892.

12 Kipnis V, Midthune D, Buckman DW, Dodd KW, Guenther PM, Krebs-Smith SM *et al.* Modeling data with excess zeros and measurement error: application to evaluating relationships between episodically consumed foods and health outcomes. *Biometrics* 2009; **65**: 1003–10.

13 Tooze JA, Midthune D, Dodd KW, Freedman LS, Krebs-Smith SM, Subar AF *et al.* A New Statistical Method for Estimating the Usual Intake of Episodically Consumed Foods with

Application to Their Distribution. *J Am Diet Assoc* 2006; **106**: 1575–1587.

14 Konstabel K, Chopra S, Ojiambo R, Muñiz-Pardos B, Pitsiladis Y. Accelerometry-Based Physical Activity Assessment for Children and Adolescents. In: *Instruments for Health Surveys in Children and Adolescents*. 2019, pp 135–173.

15 Evenson KR, Catellier DJ, Gill K, Ondrak KS, McMurray RG. Calibration of two objective measures of physical activity for children. *J Sports Sci* 2008; **26**: 1557–65.

16 Reilly JJ, Penpraze V, MacLean C, Montgomery C, Kelly LA, Paton JY *et al.* Monitoring of physical activity in young children: how much is enough? *Pediatr Exerc Sci* 2006; **18**: 483–491.

17 Bingham DD, Costa S, Clemes SA, Routen AC, Moore HJ, Barber SE. Accelerometer data requirements for reliable estimation of habitual physical activity and sedentary time of children during the early years - a worked example following a stepped approach. *J Sports Sci* 2016; **34**: 2005–10.

18 Olafsdottir S, Berg C, Eiben G, Lanfer A, Reisch L, Ahrens W *et al.* Young children's screen activities, sweet drink consumption and anthropometry: results from a prospective European study. *Eur J Clin Nutr* 2014; **68**: 223–8.

19 Bogl LH, Mehlig K, Intemann T, Masip G, Keski-Rahkonen A, Russo P *et al.* A within-sibling pair analysis of lifestyle behaviours and BMI z-score in the multi-centre I.Family study. *Nutr Metab Cardiovasc Dis NMCD* 2019; **29**: 580–589.

20 Lissner L, Lanfer A, Gwozdz W, Olafsdottir S, Eiben G, Moreno LA *et al.* Television habits in relation to overweight, diet and taste preferences in European children: the IDEFICS study. *Eur J Epidemiol* 2012; **27**: 705–15.

21 Cole TJ, Lobstein T. Extended international (IOTF) body mass index cut-offs for thinness, overweight and obesity. *Pediatr Obes* 2012; **7**: 284–94.

22 McCarthy, H., Jarrett, K., Crawley H. The development of waist circumference percentiles in British. *Eur J Clin Nutr* 2001; **55**: 902–907.

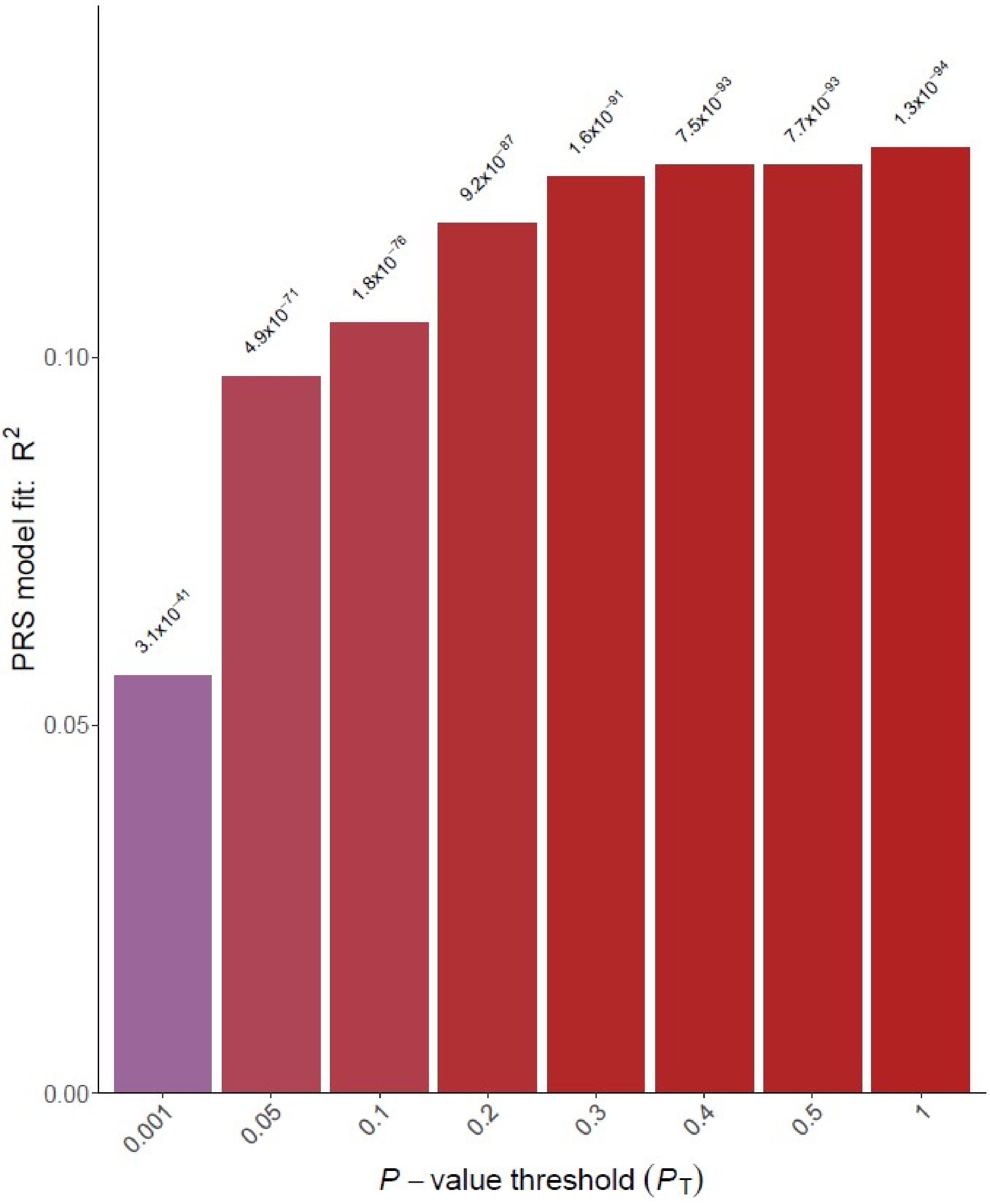

Figure S1. Association of the PRS Locke^{BT} with BMI in dependence of the p-value threshold chosen for the inclusion of SNPs. The p-values for the associations between the different PRS and BMI are stated above the bars. The highest prediction capacity (R^2) was reached when all 80,975 independent SNPs were included in the PRS (p-value threshold of 1). This PRS (Locke^{BT}; BT: best threshold) was used for all subsequent analyses.

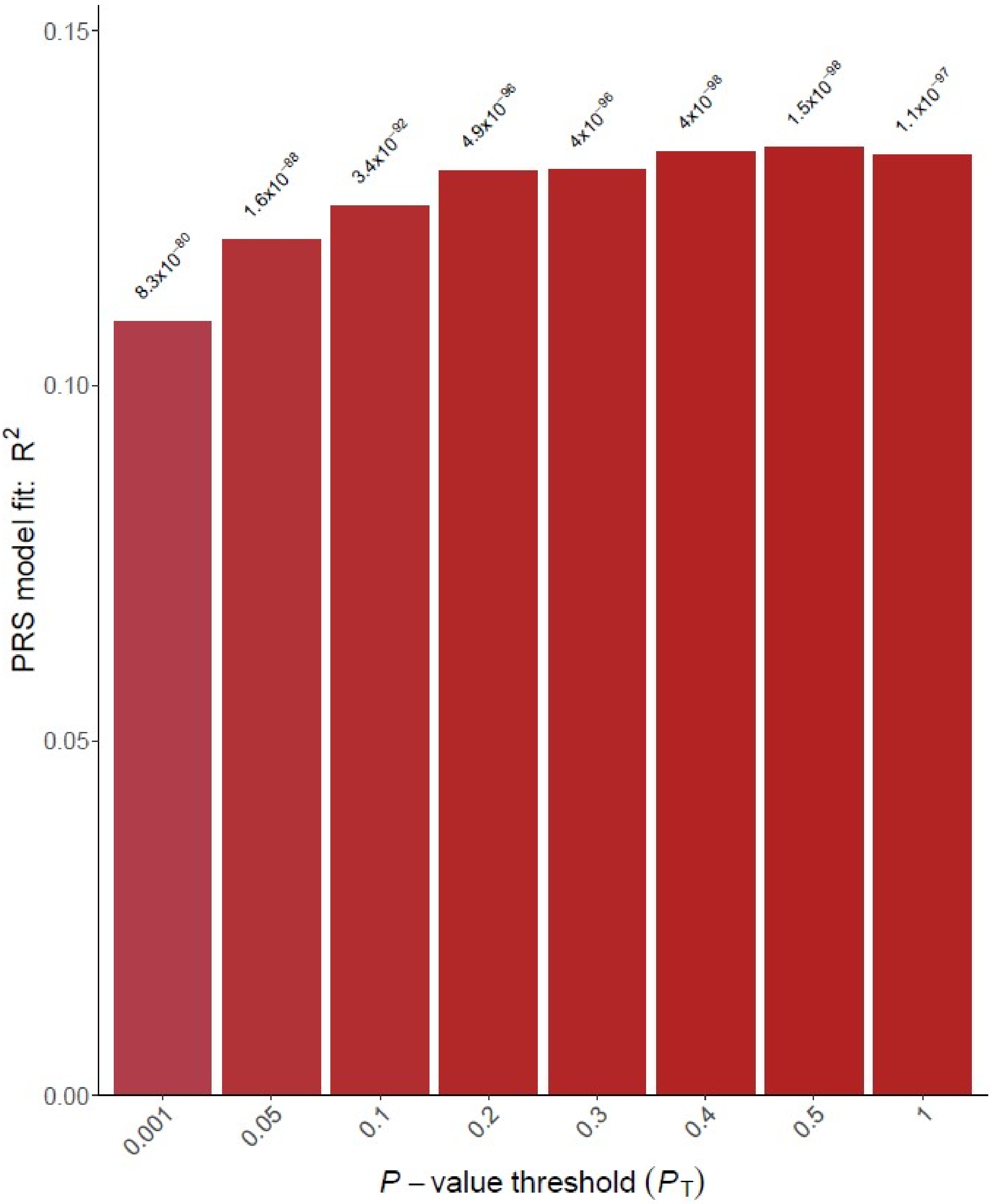

Figure S2. Association of the PRS Yengo^{BT} with BMI in dependence of the p-value threshold chosen for the inclusion of SNPs. The p-values for the associations between the different PRS and BMI are stated above the bars. The highest prediction capacity (R^2) was reached for a p-value threshold of 0.5. This PRS (Yengo^{BT}; BT: best threshold) includes 63,523 independent SNPs and was used for all subsequent analyses.

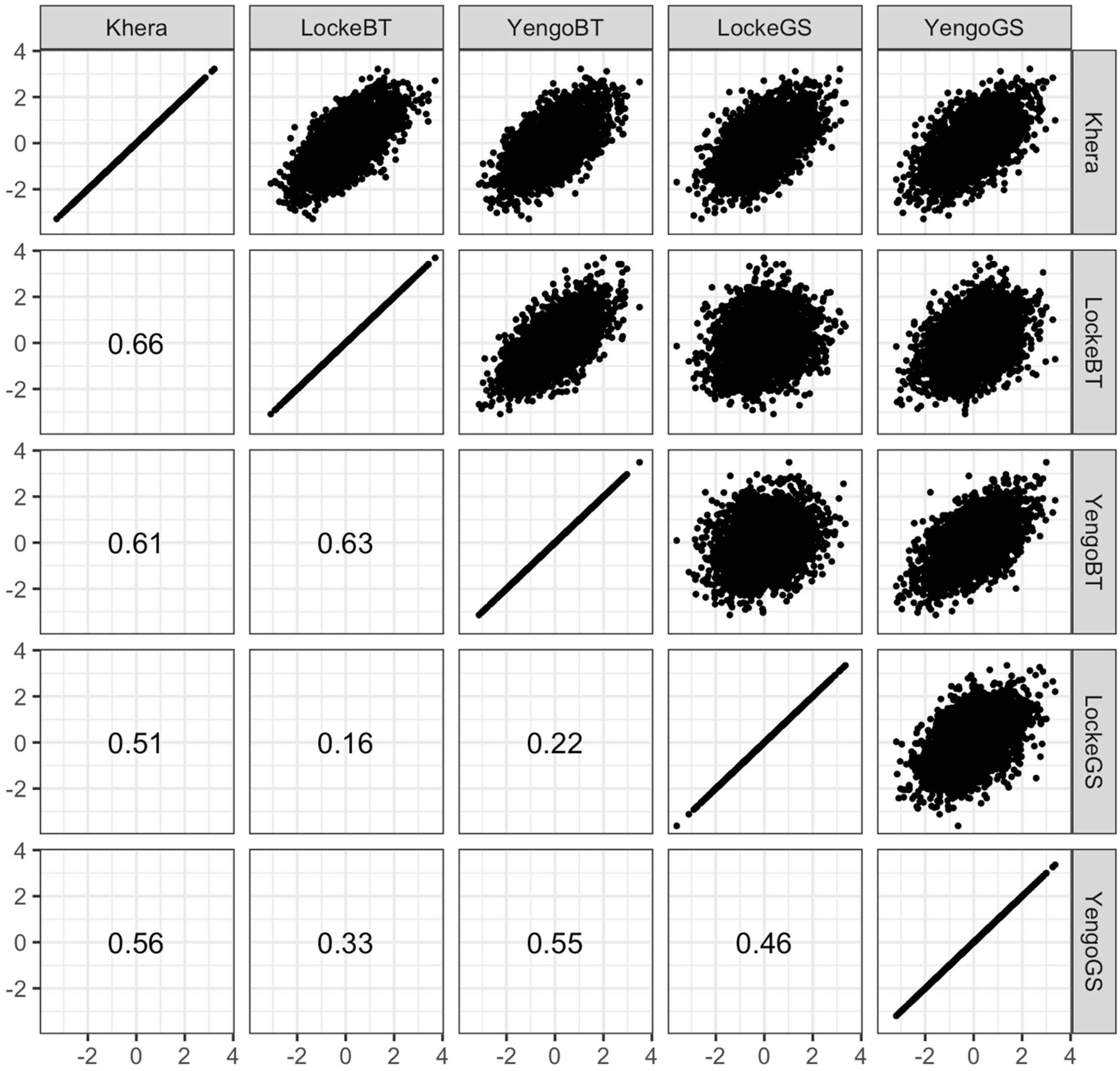

Figure S3. Spearman correlations between different PRS. The scatter plots show the pairwise comparisons of different PRS for each of the 3,098 study participants.

BT: PRSice with best p-value threshold, GS: only genome-wide significant SNPs from the discovery sample were included; discovery samples: Locke [5], Yengo [4].

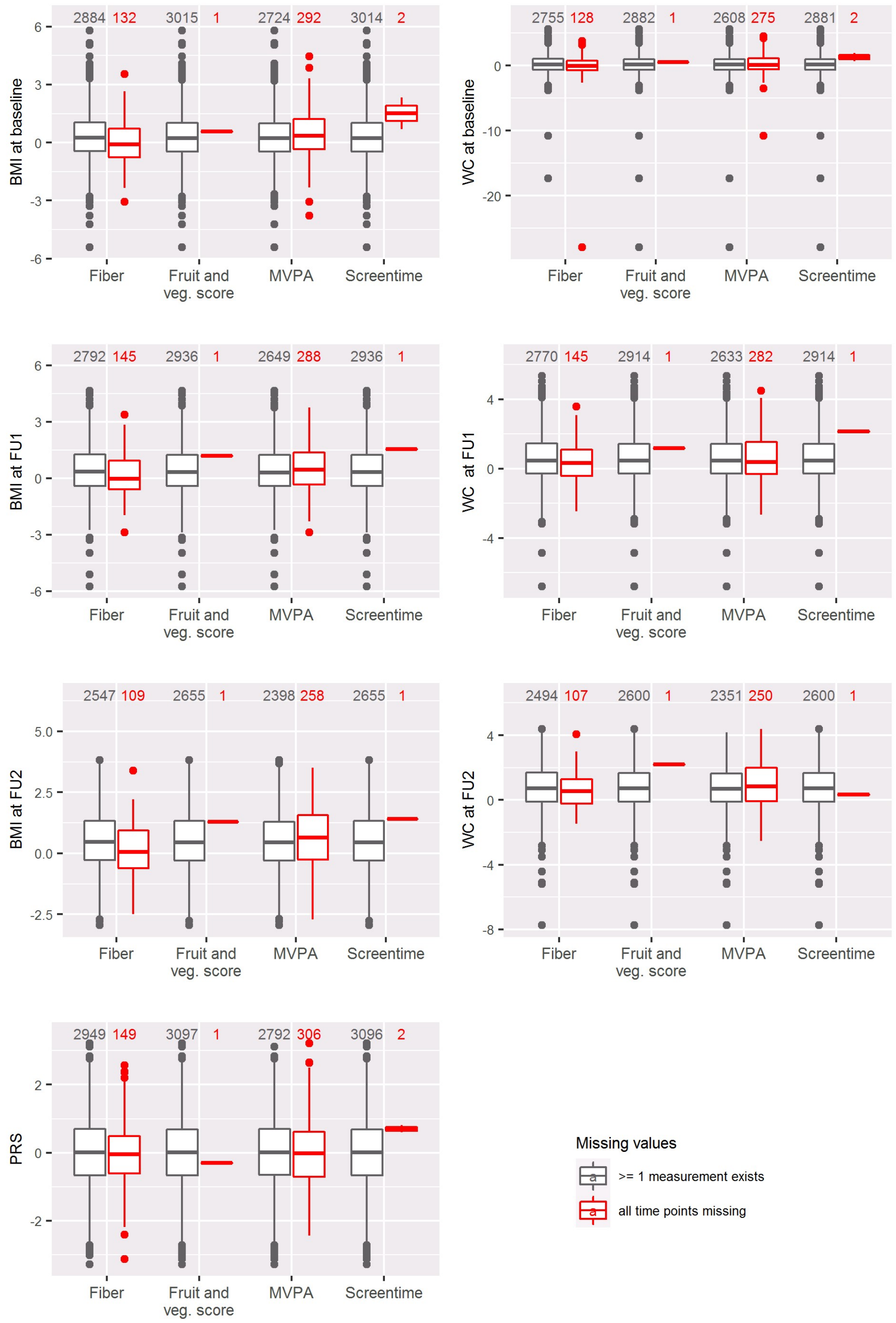

Figure S4. Boxplots showing BMI z-score and waist circumference (WC) z-score at baseline, FU1 and FU2 as well as PRS-Khera grouped according to missing data information for selected exposure variables. Numbers above boxplots denote the number of observations in the respective category.

Table S1. Associations of PRS with BMI and obesity in IDEFICS/I.Family.

			BMI			Obesity		
**Scale of PRS**	**Discovery sample (N)**	**PRS (number of SNPs)**	**Est., 95% CI**	**p-value**	**R^2**	**OR, 95% CI**	**p-value**	**AUC**
**Continuous**	Locke (~300,000)	Khera (1,079,163)	0.33 [0.30, 0.37]	7.9e-81	0.108	2.33 [2.01, 2.70]	2.0e-29	0.736
	Locke (~300,000)	Locke[BT] (80,975)	0.29 [0.25, 0.33]	6.7e-55	0.102	2.17 [1.87, 2.53]	8.9e-24	0.750
	Yengo (~700,000)	Yengo[BT] (63,523)	0.34 [0.30, 0.37]	3.7e-81	0.107	2.23 [1.93, 2.59]	2.0e-26	0.723
	Locke (~300,000)	Locke[GS] (78)	0.20 [0.17, 0.24]	4.2e-29	0.024	1.68 [1.47, 1.92]	3.6e-14	0.612
	Yengo (~700,000)	Yengo[GS] (1,302)	0.28 [0.25, 0.32]	7.2e-58	0.061	1.87 [1.62, 2.15]	8.1e-18	0.648
**Top decile**	Locke (~300,000)	Khera (1,079,163)	0.61 [0.49, 0.73]	5.4e-24	0.036	3.63 [2.57, 5.14]	2.7e-13	0.598
	Locke (~300,000)	Locke[BT] (80,975)	0.64 [0.52, 0.76]	2.1e-25	0.047	4.54 [3.25, 6.34]	8.9e-19	0.633
	Yengo (~700,000)	Yengo[BT] (63,523)	0.57 [0.45, 0.69]	9.8e-21	0.028	2.69 [1.90, 3.82]	2.9e-08	0.564
	Locke (~300,000)	Locke[GS] (78)	0.40 [0.28, 0.51]	5.9e-11	0.008	2.67 [1.84, 3.87]	2.2e-07	0.541
	Yengo (~700,000)	Yengo[GS] (1,302)	0.59 [0.47, 0.71]	1.6e-22	0.021	3.04 [2.12, 4.36]	1.7e-09	0.554

Associations adjusted for region of residence, sex, age, parental education, fruit and vegetable score. Z-scores for BMI were calculated according to [21,22]. Boys with a BMI z-score > 2.29 and girls with a BMI z-score > 2.19 were defined as obese [21,22]. BT: PRSice with best p-value threshold, GS: only genome-wide significant SNPs from the discovery sample were included; discovery samples: Locke [5], Yengo [4].

Table S2. Squared correlations (r^2) between PRS-Khera and BMI & waist circumference in dependence of age.

	BMI		Waist circumference	
**Age**	**N**	**r^2 [95% CI]**	**N**	**r^2 [95% CI]**
2	79	0.02 [0.01, 0.12]	0	NA [NA, NA]
3	381	0.03 [0.01, 0.08]	370	0.03 [0.01, 0.07]
4	541	0.05 [0.02, 0.09]	521	0.05 [0.02, 0.09]
5	712	0.08 [0.04, 0.12]	703	0.08 [0.04, 0.12]
6	937	0.08 [0.05, 0.12]	931	0.08 [0.05, 0.12]
7	1055	0.13 [0.09, 0.16]	1047	0.10 [0.07, 0.13]
8	1167	0.14 [0.10, 0.17]	1156	0.09 [0.06, 0.12]
9	1134	0.11 [0.08, 0.15]	1130	0.10 [0.07, 0.13]
10	930	0.14 [0.10, 0.18]	919	0.11 [0.08, 0.15]
11	377	0.14 [0.08, 0.21]	367	0.14 [0.08, 0.21]
12	382	0.15 [0.09, 0.22]	375	0.11 [0.06, 0.18]
13	599	0.12 [0.08, 0.18]	576	0.08 [0.05, 0.13]
14	304	0.18 [0.11, 0.27]	295	0.14 [0.08, 0.22]
15	10	NA [NA, NA]	9	NA [NA, NA]
16	1	NA [NA, NA]	1	NA [NA, NA]

Squared correlations could not be calculated for ≥15-year old children due to the small sample size in these age groups. Waist circumference was not measured in 2-year old children.

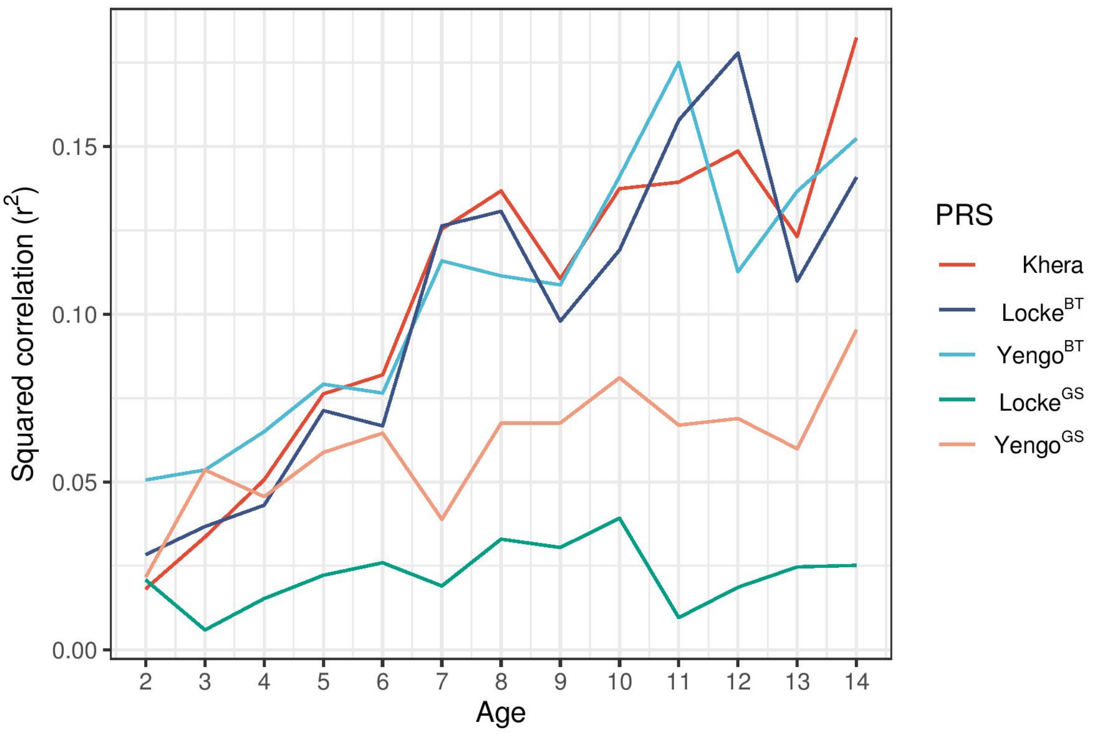

Figure S5. Squared correlation (r^2) of the different PRS with BMI in dependence of age. Squared correlations could not be calculated for ≥15-year old children due to the small sample size in these age groups (see Table S2).
BT: PRSice with best p-value threshold, GS: only genome-wide significant SNPs from the discovery sample were included; discovery samples: Locke [5], Yengo [4].

Table S3. Squared correlations (r^2) [95% CI] between the different PRS and BMI in dependence of age.

Age	N	Khera	Locke^{BT}	Yengo^{BT}	Locke^{GS}	Yengo^{GS}
2	79	0.02 [0.01, 0.12]	0.03 [0.00, 0.14]	0.05 [0.00, 0.18]	0.02 [0.01, 0.13]	0.02 [0.01, 0.13]
3	381	0.03 [0.01, 0.08]	0.04 [0.01, 0.08]	0.05 [0.02, 0.11]	0.01 [0.00, 0.03]	0.05 [0.02, 0.11]
4	541	0.05 [0.02, 0.09]	0.04 [0.02, 0.08]	0.07 [0.03, 0.11]	0.02 [0.00, 0.04]	0.05 [0.02, 0.09]
5	712	0.08 [0.04, 0.12]	0.07 [0.04, 0.11]	0.08 [0.05, 0.12]	0.02 [0.01, 0.05]	0.06 [0.03, 0.10]
6	937	0.08 [0.05, 0.12]	0.07 [0.04, 0.10]	0.08 [0.05, 0.11]	0.03 [0.01, 0.05]	0.06 [0.04, 0.10]
7	1055	0.13 [0.09, 0.16]	0.13 [0.09, 0.17]	0.12 [0.08, 0.15]	0.02 [0.01, 0.04]	0.04 [0.02, 0.06]
8	1167	0.14 [0.10, 0.17]	0.13 [0.10, 0.17]	0.11 [0.08, 0.15]	0.03 [0.02, 0.06]	0.07 [0.04, 0.10]
9	1134	0.11 [0.08, 0.15]	0.10 [0.07, 0.13]	0.11 [0.08, 0.14]	0.03 [0.01, 0.05]	0.07 [0.04, 0.10]
10	930	0.14 [0.10, 0.18]	0.12 [0.08, 0.16]	0.14 [0.10, 0.18]	0.04 [0.02, 0.07]	0.08 [0.05, 0.12]
11	377	0.14 [0.08, 0.21]	0.16 [0.10, 0.23]	0.18 [0.11, 0.25]	0.01 [0.00, 0.04]	0.07 [0.03, 0.12]
12	382	0.15 [0.09, 0.22]	0.18 [0.11, 0.25]	0.11 [0.06, 0.18]	0.02 [0.00, 0.05]	0.07 [0.03, 0.12]
13	599	0.12 [0.08, 0.18]	0.11 [0.07, 0.16]	0.14 [0.09, 0.19]	0.02 [0.01, 0.05]	0.06 [0.03, 0.10]
14	304	0.18 [0.11, 0.27]	0.14 [0.08, 0.22]	0.15 [0.08, 0.23]	0.03 [0.00, 0.07]	0.10 [0.04, 0.17]
15	10	NA [NA, NA]	NA [NA, NA]	NA [NA, NA]	NA [NA, NA]	NA [NA, NA]
16	1	NA [NA, NA]	NA [NA, NA]	NA [NA, NA]	NA [NA, NA]	NA [NA, NA]

Squared correlations could not be calculated for ≥15-year old children due to the small sample size in these age groups.
BT: PRSice with best p-value threshold, GS: only genome-wide significant SNPs from the discovery sample were included; discovery samples: Locke [5], Yengo [4].

Table S4. Interactions between PRS-Khera and parental education for BMI and waist circumference.

A) BMI (n = 8223)

**Sociodemographic variable**	**Category**	**Est., 95% CI**	**p-value**	**q-value**
Sex	Male	0.33 [0.28,0.37]	Reference	
	Female	0.34 [0.29,0.39]	0.6748	0.7592
Parental education	Low	0.48 [0.38,0.59]	**0.0012**	**0.0106**
	Medium	0.35 [0.31,0.39]	0.0645	0.1160
	High	0.30 [0.26,0.34]	Reference	
Region	Central	0.29 [0.23,0.34]	Reference	
	North	0.32 [0.25,0.39]	0.4779	0.7169
	South	0.40 [0.34,0.45]	**0.0066**	**0.0246**

B) Waist circumference (n = 8027)

**Sociodemographic variable**	**Category**	**Est., 95% CI**	**p-value**	**q-value**
Sex	Male	0.34 [0.29,0.39]	Reference	
	Female	0.37 [0.32,0.43]	0.3842	0.4940
Parental education	Low	0.53 [0.40,0.67]	**0.0051**	**0.0232**
	Medium	0.37 [0.31,0.42]	0.2579	0.3869
	High	0.33 [0.27,0.38]	Reference	
Region	Central	0.30 [0.24,0.36]	Reference	
	North	0.40 [0.32,0.47]	**0.0471**	0.1059
	South	0.39 [0.33,0.46]	**0.0380**	0.1059

Associations between PRS and obesity are shown in different strata (beta estimates and 95% confidence intervals). P-values are given for the test of deviations of the association between PRS and obesity in one subgroup in comparison to the reference category (interaction). q-values were adjusted according to the number of tested environmental factors using the false-discovery rate (FDR).

Table S5. Interaction between PRS-Khera and lifestyle factors for BMI and waist circumference.

A) BMI

**Environmental factor**	**n**	**Est., 95% CI**	**p-value**	**q-value**
Fruit and vegetable score	8223	0.00 [-0.02, 0.02]	0.9058	0.9058
Fiber intake	5963	-0.02 [-0.04,-0.01]	**0.0082**	**0.0246**
MVPA	5016	-0.01 [-0.07, 0.04]	0.6079	0.7592
Screen time	8018	0.02 [0.00, 0.03]	**0.0185**	**0.0416**

B) Waist circumference

**Environmental factor**	**n**	**Est., 95% CI**	**p-value**	**q-value**
Fruit and vegetable score	8027	-0.04 [-0.33, 0.25]	0.7792	0.7792
Fiber intake	5846	-0.03 [-0.06,-0.01]	**0.0040**	**0.0232**
MVPA	4943	-0.05 [-0.12, 0.03]	0.2014	0.3625
Screen time	7825	0.00 [-0.01, 0.02]	0.6391	0.7190

Beta estimates, 95% confidence intervals and p-values are given for the interaction between the PRS and each environmental factor with BMI. q-values were adjusted according to the number of tested environmental factors using the false-discovery rate (FDR).

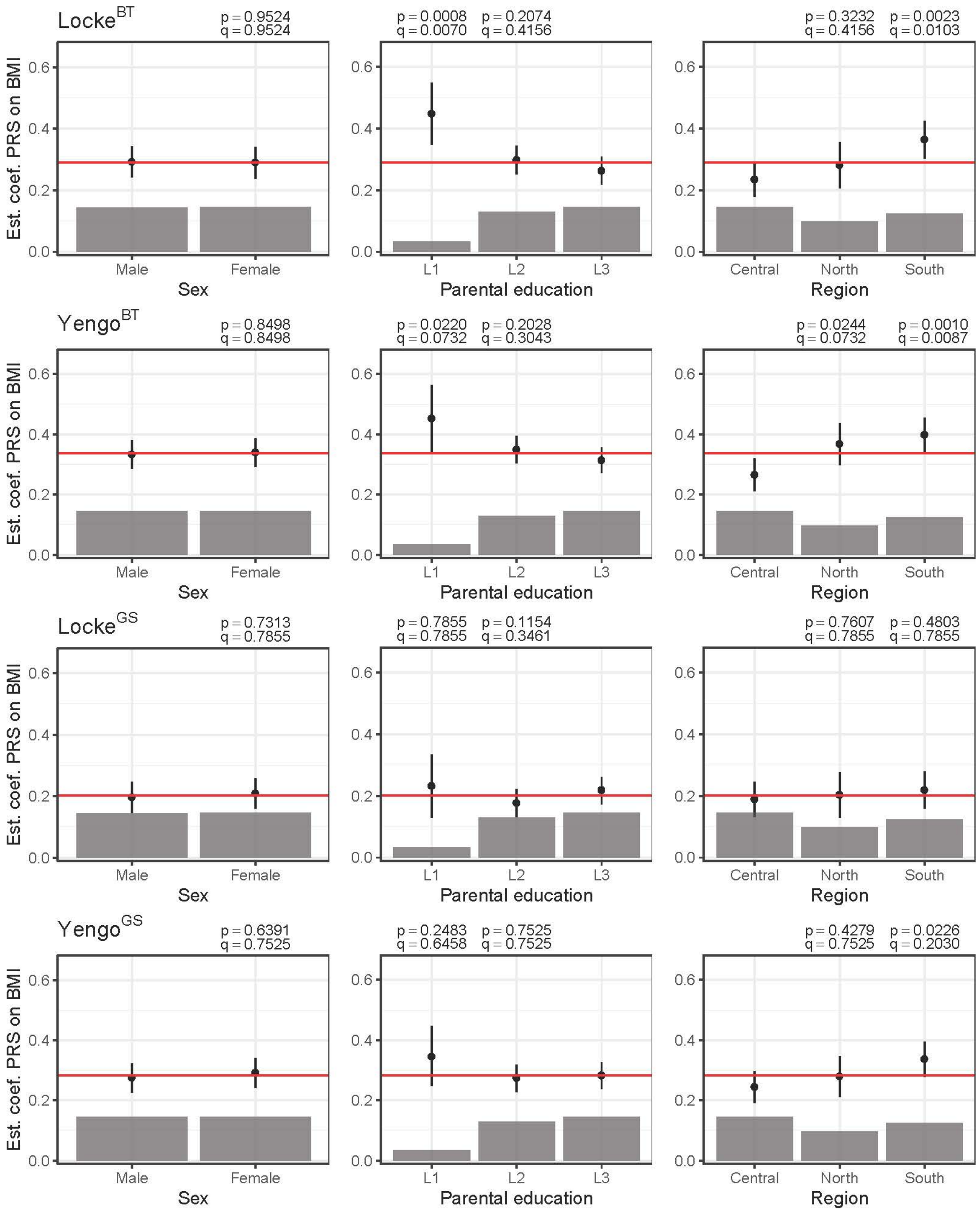

Figure S6. Interactions of Locke^{BT}, Yengo^{BT}, Locke^{GS} and Yengo^{GS} with sociodemographic factors for BMI (n = 8223). Associations between PRS and obesity are shown in different strata (beta estimates and 95% confidence intervals) as well as in the whole study population (red line). The grey boxes show the distribution of the sociodemographic factors.
BT: PRSice with best p-value threshold, GS: only genome-wide significant SNPs from the discovery sample were included; discovery samples: Locke [5], Yengo [4].

Table S6. Interactions between different PRS and sociodemographic variables for BMI (n = 8223).

A) Locke^{BT}

**Sociodemographic variable**	**Category**	**Est., 95% CI**	**p-value**	**q-value**
Sex	Male	0.29 [0.24,0.34]	Reference	
	Female	0.29 [0.24,0.34]	0.9524	0.9524
Parental education	Low	0.45 [0.35,0.55]	**0.0008**	**0.0070**
	Medium	0.30 [0.25,0.35]	0.2074	0.4156
	High	0.26 [0.22,0.31]	Reference	
Region	Central	0.24 [0.18,0.29]	Reference	
	North	0.28 [0.21,0.35]	0.3232	0.4156
	South	0.36 [0.30,0.43]	**0.0023**	**0.0103**

B) Yengo^{BT}

**Sociodemographic variable**	**Category**	**Est., 95% CI**	**p-value**	**q-value**
Sex	Male	0.33 [0.28,0.38]	Reference	
	Female	0.34 [0.29,0.39]	0.8498	0.8498
Parental education	Low	0.45 [0.34,0.57]	**0.0220**	0.0732
	Medium	0.35 [0.30,0.39]	0.2028	0.3043
	High	0.31 [0.27,0.36]	Reference	
Region	Central	0.27 [0.21,0.32]	Reference	
	North	0.37 [0.30,0.44]	**0.0244**	0.0732
	South	0.40 [0.34,0.46]	**0.0010**	**0.0087**

C) Locke^{GS}

**Sociodemographic variable**	**Category**	**Est., 95% CI**	**p-value**	**q-value**
Sex	Male	0.20 [0.15,0.25]	Reference	
	Female	0.21 [0.16,0.26]	0.7313	0.7855
Parental education	Low	0.23 [0.13,0.34]	0.7855	0.7855
	Medium	0.18 [0.13,0.22]	0.1154	0.3461
	High	0.22 [0.18,0.26]	Reference	
Region	Central	0.19 [0.14,0.24]	Reference	
	North	0.20 [0.13,0.27]	0.7607	0.7855
	South	0.22 [0.16,0.28]	0.4803	0.7855

D) Yengo^{GS}

Sociodemographic variable	Category	Est., 95% CI	p-value	q-value
Sex	Male	0.27 [0.23,0.32]	Reference	
	Female	0.29 [0.24,0.34]	0.6391	0.7525
Parental education	Low	0.35 [0.24,0.44]	0.2483	0.6458
	Medium	0.27 [0.23,0.32]	0.7525	0.7525
	High	0.28 [0.24,0.33]	Reference	
Region	Central	0.24 [0.19,0.30]	Reference	
	North	0.28 [0.21,0.35]	0.4279	0.7525
	South	0.34 [0.28,0.40]	**0.0226**	0.2030

BT: PRSice with best p-value threshold, GS: only genome-wide significant SNPs from the discovery sample were included; discovery samples: Locke [5], Yengo [4].

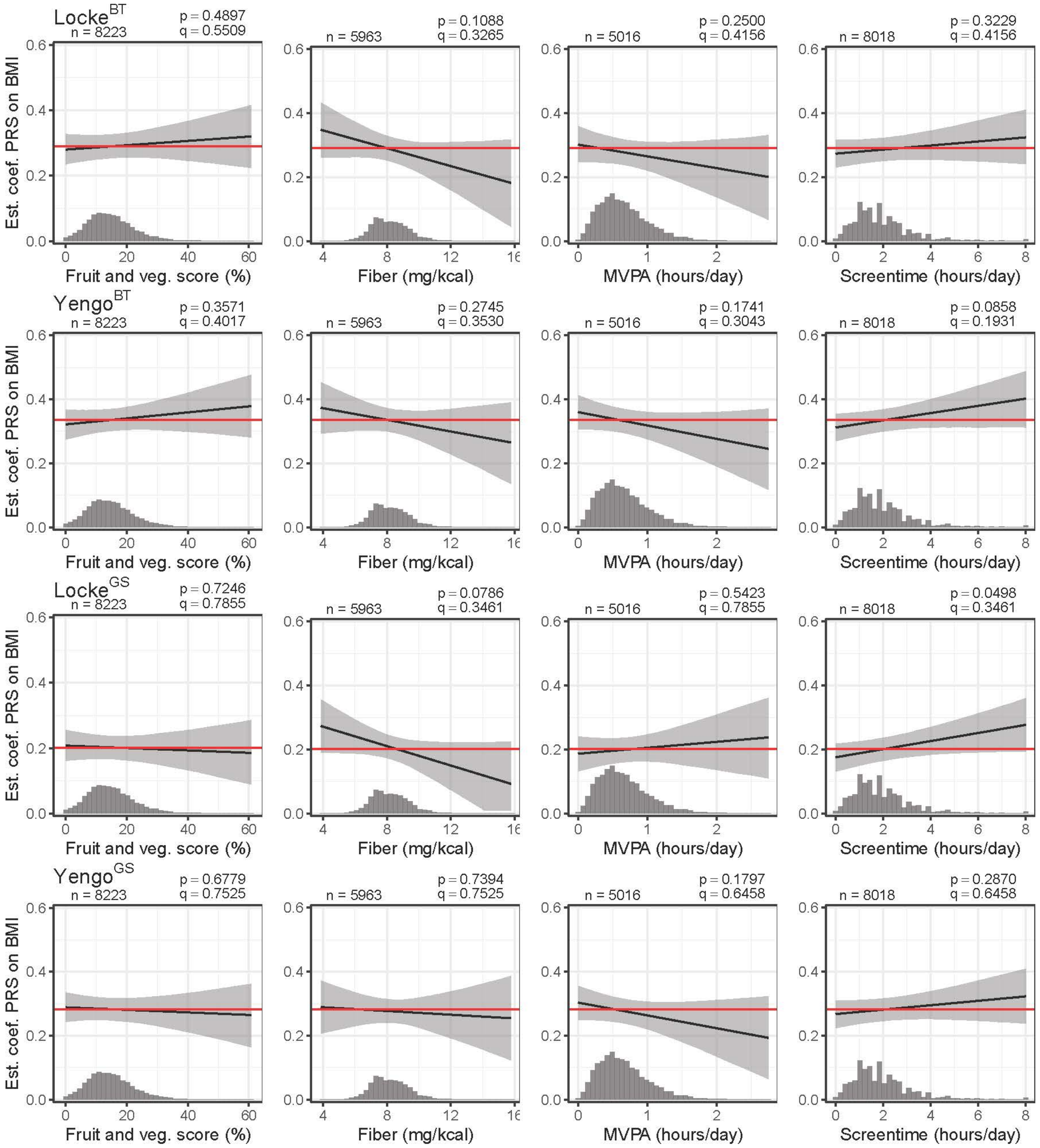

Figure S7. Interaction of Locke^{BT}, Yengo^{BT}, Locke^{GS} and Yengo^{GS} with lifestyle factors for BMI. Associations between PRS and obesity are shown in dependence of the PRS (beta estimates and 95% confidence intervals) as well as in the whole study population (red line). The distributions of the lifestyle factors are shown in histograms.

BT: PRSice with best p-value threshold, GS: only genome-wide significant SNPs from the discovery sample were included; discovery samples: Locke [5], Yengo [4].

Table S7. Interactions between different PRS and lifestyle factors for BMI.

A) Locke^{BT}

**Environmental factor**	**n**	**Est., 95% CI**	**p-value**	**q-value**
Fruit and vegetable score	8223	0.01 [-0.01, 0.03]	0.4897	0.5509
Fiber intake	5963	-0.01 [-0.03, 0.00]	0.1088	0.3265
MVPA	5016	-0.04 [-0.10, 0.03]	0.2500	0.4156
Screen time	8018	0.01 [-0.01, 0.02]	0.3229	0.4156

B) Yengo^{BT}

**Environmental factor**	**n**	**Est., 95% CI**	**p-value**	**q-value**
Fruit and vegetable score	8223	0.01 [-0.01, 0.03]	0.3571	0.4017
Fiber intake	5963	-0.01 [-0.03, 0.01]	0.2745	0.3530
MVPA	5016	-0.04 [-0.10, 0.02]	0.1741	0.3043
Screen time	8018	0.01 [0.00, 0.02]	0.0858	0.1931

C) Locke^{GS}

**Environmental factor**	**n**	**Est., 95% CI**	**p-value**	**q-value**
Fruit and vegetable score	8223	0.00 [-0.02, 0.02]	0.7246	0.7855
Fiber intake	5963	-0.02 [-0.03, 0.00]	0.0786	0.3461
MVPA	5016	0.02 [-0.04, 0.08]	0.5423	0.7855
Screen time	8018	0.01 [0.00, 0.03]	**0.0498**	0.3461

D) Yengo^{GS}

**Environmental factor**	**n**	**Est., 95% CI**	**p-value**	**q-value**
Fruit and vegetable score	8223	0.00 [-0.02, 0.02]	0.6779	0.7525
Fiber intake	5963	0.00 [-0.02, 0.01]	0.7394	0.7525
MVPA	5016	-0.04 [-0.10, 0.02]	0.1797	0.6458
Screen time	8018	0.01 [-0.01, 0.02]	0.2870	0.6458

BT: PRSice with best p-value threshold, GS: only genome-wide significant SNPs from the discovery sample were included; discovery samples: Locke [5], Yengo [4].
